# Leukotriene B_4_ Loaded in Microspheres Inhibits Osteoclast Differentiation and Activation

**DOI:** 10.1590/0103-6440202204827

**Published:** 2022-10-21

**Authors:** Francine Lorencetti-Silva, Maya Fernanda Manfrin Arnez, João Pedro de Queiroz Thomé, Marcio Santos de Carvalho, Fabrício Kitazono de Carvalho, Alexandra Mussolino de Queiroz, Lúcia Helena Faccioli, Francisco Wanderley Garcia Paula-Silva

**Affiliations:** 1 Departamento de Clínica Infantil, Faculdade de Odontologia de Ribeirão Preto, Universidade de São Paulo, Ribeirão Preto, São Paulo, Brasil.; 2 Universidade de Rio Verde, Rio Verde, Goiás, Brasil.; 3 Faculdade de Ciências da Saúde de Barretos Dr. Paulo Prata, Barretos, SP, Brazil.; 4 Departamento de Análises Clínicas, Toxicológicas e Bromatológicas da Faculdade de Ciências Farmacêuticas de Ribeirão Preto, Universidade de São Paulo, Ribeirão Preto, São Paulo, Brazil.

**Keywords:** Osteoclast, leukotriene B4, macrophages, differentiation, activation, microsphere

## Abstract

To investigate osteoclast formation *in vivo* and if leukotriene B_4_ (LTB_4_) loaded in microspheres (MS) could be used as a therapeutical strategy to promote a sustained delivery of the mediator and prevent osteoclast differentiation. Methods: *In vivo*, apical periodontitis was induced in mice to investigate osteoclast differentiation and signaling in absence of 5-lipoxygenase (5-LO). *In vitro,* LTB_4_-MS were prepared using an oil-in-water emulsion solvent extraction-evaporation process. Characterization and efficiency of LTB_4_ encapsulation were investigated. J774A.1 macrophages were cultured in the presence of monocyte colony-stimulating factor (M-CSF) and ligand for receptor activator of nuclear factor kappa B (RANKL) and then stimulated with LTB_4_-MS. Cytotoxicity, *in vitro* MS-LTB_4_ uptake, osteoclast formation and gene expression were measured. Results: We found that 5-LO negatively regulates osteoclastic formation *in vivo* during apical periodontitis development. *In vitro*, LTB_4_-MS were up-taken by macrophages and were not cytotoxic to the cells. LTB_4_-MS inhibited osteoclast formation and the synthesis of osteoclastogenic genes *Acp5*, *Mmp9*, *Calcr* and *Ctsk.* LTB_4_-MS inhibited differentiation of macrophages into an osteoclastic phenotype and cell activation under M-CSF and RANKL stimulus.

## Resumo

O objetivo deste trabalho foi Investigar a formação de osteoclastos *in vivo* e se o leucotrieno B_4_ (LTB_4_) incorporado em microesferas (MS) poderia ser usado como estratégia terapêutica para promover uma entrega sustentada do mediador e prevenir a diferenciação dos osteoclastos. Métodos: In vivo, a periodontite apical foi induzida em camundongos para investigar a diferenciação e sinalização de osteoclastos na ausência de 5-lipoxigenase (5-LO). In vitro, LTB_4_-MS foi preparado usando um processo de evaporação e extração de solvente de emulsão de óleo em água. A caracterização e a eficiência do encapsulamento do LTB_4_ foram investigadas. Macrófagos J774A.1 foram cultivados na presença de fator estimulador de colônia de monócitos (M-CSF) e ligante para o receptor ativador do fator nuclear kappa B (RANKL) e, então, estimulados com LTB4-MS. Citotoxicidade, captação *in vitro* de MS-LTB_4_, formação de osteoclastos e expressão gênica foram avaliadas. Resultados: A via 5-LO regula negativamente a formação de osteoclastos *in vivo* durante o desenvolvimento da periodontite apical. *In vitro*, LTB_4_-MS foram fagocitadas pelos macrófagos e não foram citotóxicos para as células. LTB_4_-MS inibiu a formação de osteoclastos e a síntese dos genes pró-osteoclastogênicos *Acp5*, *Mmp9*, *Calcr* e *Ctsk*. Conclusões: LTB_4_-MS inibiu a diferenciação de macrófagos em um fenótipo osteoclástico e a ativação celular sob estímulo de M-CSF e RANKL.

## Introduction

Bone resorption resulting from periapical inflammation is a process that involves a series of steps, starting with the recruitment of monocytes from hematopoietic lineage, followed by osteoclast differentiation and maturation, ultimately resulting in bone loss ^1^ ^,^ ^2^ ^,^ ^3^. Several cytokines and growth factors are essential for the differentiation and maturation of osteoclasts, including interleukins (IL-1α, IL-6, IL-11, IL-15, IL-17 and IL-18), tumor necrosis factor-alpha (TNF-α), monocyte colony-stimulating factors (M-CSF, CSF2 and CSF3), prostaglandins and matrix metalloproteinases ^1^ ^,^ ^3^ ^,^ ^4^. These factors stimulate osteoclast progenitor cells or in the regulation of a paracrine system that includes the molecules RANK (Receptor activator of nuclear factor kappa B), ligand for RANK (RANKL) and osteoprotegerin (OPG) ^1^ ^,^ ^5^. Signaling between cells of the immune system, cells of the hematopoietic lineage and resident cells might result in degradation of organic and inorganic matrices of bone tissue, depending on the nature of the mediators involved in the process ^1^ ^,^ ^6^.

Soluble mediators RANKL and OPG regulate the canonical process of osteoclastogenesis. RANKL, a soluble mediator member of the tumor necrosis factor superfamily, induces osteoclast activation and fusion when it binds to RANK ^7^. RANKL induce the expression of several osteclastogenic lineage genes including tartrate-resistant acid phosphatase (*Acp5*), cathepsin K (*Ctsk*), matrix-9 metalloproteinase (*Mmp9*) and the calcitonin receptor (*Calcr*), thus leading to maturation of these cells ^1^ ^,^ ^5^. Effects of RANKL are blocked by soluble receptors, such as osteoprotegerin, secreted by osteoblasts, which inhibits osteoclast differentiation ^8^.

Lipid mediators derived from arachidonic acid are widely studied in inflammation and inflammatory cell differentiation ^9^ ^,^ ^10^ ^,^ ^11^ ^,^ ^12^. Previous studies have demonstrated that bacterial lipopolysaccharide (LPS) or contamination of dental root canals induced periapical inflammation and the expression of the *Alox5* gene, responsible for encoding the 5-lipoxygenase (5-LO) enzyme ^13^ ^,^ ^14^ ^,^ ^15^. Gene disruption ^15^ or pharmacological blockage ^13^ ^)^ of 5-LO resulted in increased expression of molecules regulators of osteoclast formation, compared to wild-type animals indicating a role of 5-LO arachidonic pathway on osteoclastogenesis. Nonetheless, the role of lipid mediators generated upon 5-LO activation were not further investigated

Leukotriene B4 (LTB_4_) is a lipid mediator of inflammation produced by leukocytes and derived from arachidonic acid by the 5-LO pathway ^16^ ^,^ ^17^ ^,^ ^18^. LTB4 is expressed by a variety of phagocytic cells, including neutrophils, monocytes, macrophages and mast cells ^16^. After its release, it induces an immunoinflammatory response and chemotaxis in order to enhance the elimination of pathogenic microorganisms or injurious stimuli ^18^ ^,^ ^19^. These actions occur after the binding of LTB4 in its receptors with high-affinity (BLT1) or low-affinity (BLT2). Both LTB4 receptors are G protein-coupled receptors (GPRCs) and their action causes a disturbance in the cell membrane that increases the intracellular calcium (Ca2+) levels ^16^ ^,^ ^18^. While Ca2+ release leads to activation of nuclear factor of activated T-cells c1 (NFATc1) which increases osteoclast activity and intensifies the bone resorption process ^16^, BLT1 activation might suppress osteoclast activation via via NF-kB and Ca2^+^ signaling ^20^. BLT1 deficient osteoclasts produce higher amounts of mRNA of the osteoclast-related genes *Acp5*, *Ctsk*, and *Mmp9*, which facilitate bone resorption. Furthermore, *in vitro* studies showed that LTB4 elevated levels are associated with osteoclastogenesis ^16^. This production was lower than RANKL stimulation and required longer time ^16^. Thus, the objective of this study was to investigate if sustained delivery of LTB4-loaded microspheres (MS) could be used as a therapeutic strategy to prevent osteoclast differentiation.

## Methods

### Apical periodontitis induction

Experimental protocols in this study followed The National Institutes of Health Guidelines for the Care and Use of Laboratory Animals and were approved by the Institutional Animal Care and Use Committee (IACUC) from the University of São Paulo (USP; process# 12.1.60.53.8). Sample size calculation was determined based on the results of a previous study ^13^, using a statistical power of 80%. Twenty-four knockout mice for the 5-LO enzyme (129-Alox5^-/-^; The Jackson Laboratory, Bar Harbor, ME, USA) and 24 129 wild-type 6-8-week-old littermates mice were used. For the operative procedures, the animals were anesthetized with ketamine hydrochloride (150 mg/kg, ketamine 10%, Agener União Química Farmacêutica Nacional S/A, Embu-Guaçu, SP) and xylazine (7.5 mg/kg, Dopaser, Laboratorios Calier S/A, Barcelona, Spain), anesthesia was sustained during the entire operative procedure.

To induce apical periodontitis, the coronary opening was performed by the occlusal face in the lower and upper right first molars using a 1011 spherical diamond tip (KG Sorensen Ind. Com. Ltda., Barueri, SP). Next, the root canals were located through a type K file number 06 (Les Fils d'Auguste Maillefer S / A, Switzerland) and the dental root pulp was removed (n= 6 teeth). Root canals were exposed to oral cavity, as previously described ^13^ ^,^ ^15^. Healthy molar teeth on the left side from the same mice, without pulp exposure, were used as controls (n= 6 teeth). Mice were monitored by a veterinarian throughout the entire experimental period. At 14 and 28 days after root canal contamination, the animals were euthanized by i.m. anesthetic overdose and teeth were collected for further analysis.

### Tissue processing and histoenzymology

The lower molars were used for histological processing. Jaws were dissected and removed with surgical scissors. Blocks containing tooth and bone were fixed in 10% buffered formalin for 24 h at room temperature and demineralized in 10% EDTA (Merck S.A. Chemical Industries, Rio de Janeiro, RJ) for approximately 21 days. After demineralization, the pieces were submitted to routine histological processing, washed in running water for 24 hours, dehydrated in increasing concentrations of alcohol, diaphanized in xylol, and embedded in paraffin. The blocks were sectioned longitudinally, in a bucco-lingual direction; to obtain cuts with a thickness of 5 µm. Sections were prepared for tartrate-resistant acid phosphatase (TRAP) enzyme for histoenzymological analysis of osteoclasts.

The deparaffinized tissue sections were incubated in a solution containing 8 mg of naphthol AS-MX di-sodium phosphate (Sigma-Aldrich) in 500 μL of NN-dimethylformamide followed by the addition of 50 mL of a 0.2 mol buffer solution/L sodium acetate (pH 5.0) containing 70 mg of Fast Red ITR (Sigma-Aldrich). Subsequently the sodium tartrate dihydrate substrate (50 mmol/L) was added to the solution and incubated at 37°C for 12 h. Subsequently, the slides were washed in distilled water and stained with hematoxylin. Quantitative analysis of the number of osteoclasts positive for the TRAP enzyme was performed taking into consideration the total number of osteoclasts per apical periodontitis lesion. The groups were compared by means of two-way ANOVA followed by the Sidak post-test (α = 0.05).

### Preparation of microspheres

Microspheres (MS) were prepared as a pharmacological strategy using an oil-in-water emulsion solvent extraction-evaporation process ^21^ ^,^ ^22^. Briefly, LTB_4_ (CAYM-14010; Cayman Chemical Company, Michigan, USA) was dissolved in absolute ethanol (100 µg/mL). Then, 0.3 mL of the organic phase, equivalent to 3× 10^-5^M of the LTB_4_ solution was added to 10 mL of methylene chloride supplemented with 30 mg of 50:50 poly (lactic-co-glycolic acid) (PLGA) (Boehringer Ingelheim, Germany). Next, 40 mL of 3% polyvinyl alcohol (3% w/v PVA) (Sigma-Aldrich CO., St. Louis, MO, USA) were added and the mixture was mechanically stirred at 600 rpm for 4 h (RW-20; Ika®-Werke GmbH & CO. KG, Staufen, Germany). Microspheres were washed (3x) with deionized water (Milli-Q®, Merck Millipore, Darmstadt, Germany), lyophilized, and stored at -20 °C until use. LTB_4_ concentration were based on previous studies (18, 21, 22).

### Sterility and LPS contamination tests

A sterility test was performed in which small microsphere aliquots were diluted in 500 µL of 1x PBS (phosphate buffered saline) and 100 µL of solution was spread on Brain Heart Infusion (BHI)-Agar medium and kept in an incubator at 37^o^ C for 24 h to detect microbial contamination.

Microspheres were tested for LPS contamination using the Limulus Amebocyte Lysate (LAL) QCL-1000™ kit (Lonza Walkersville, Inc., Olten, Switzerland) according to the manufacturer’s instructions. To obtain the standard curve, the serial dilution regime was adopted, starting from 1.0 EU / mL of *E. coli* endotoxin 0111: B4 (E50-640). Optical density was analyzed at a wavelength of 405 ηm in a μQuantTM spectrophotometer (BioTek® Instruments Inc., Winooski, USA), using the KC4^TM^ Data Analysis Software (BioTek® Instruments Inc.), to determine the concentration of endotoxin units in each mL of solution containing microspheres (EU / mL).

### Characterization of microspheres

Size distribution of MS was determined using a LS 13 320 Laser Diffraction Particle Size Analyzer (Beckman Coulter, USA). Samples (1 mg) of either unloaded-MS or LTB_4_ -loaded MS was dispersed in 0.4 mL of purified sterile water and then analyzed at 25 °C. Zeta potential of MS was determined using a Zetasizer Nano (Malvern Instruments, England). Each sample was prepared dispersing 1 mg of unloaded-MS or LTB_4_-loaded MS in 0.4 mL of purified water containing 10 mM NaCl and then analyzed at 25 °C. Morphology of MS samples was assessed by scanning electron microscopy (SEM) using a FEI Inspect S 50 scanning microscope (FEI; Oregon, USA).

### Efficiency of LTB_4_ encapsulation in MS

For calculation of encapsulation efficiency, samples of LTB_4_- loaded MS (4 mg) were dissolved in 1 mL of acetonitrile/ethanol (7:3 v/v), to disrupt the MS structure. The solvent was then evaporated off in a vacuum concentrator centrifuge for 4 h, and the residue was reconstituted in 100*μ*L of methanol. The supernatants were transferred to appropriate vials for determination of the concentration of LTB_4_ by a competition enzyme immunoassay, according to manufacturer's instructions (EIA, Amershan Biosciences, Piscataway, NJ, USA). Quantification was accomplished using calibration curve containing LTB_4_ synthetic standards (Cayman Chemical, Ann Arbor, MI, USA).

### In vitro LTB_4_ release assay

The release kinetics of LTB_4_ from LTB_4_-MS were monitored *in vitro*. LTB_4_ (4 mg) was suspended in 1 mL of PBS/ethanol (50:50, v/v), pH 7.4, and incubated at 37 °C on a rotating incubator. At each time point 6, 12, 18, 24, 30, 36, 42, 48 and 54 h of rotation, the suspension was centrifuged and the supernatant was collected for assay of LTB_4_ concentration, then 1 mL of fresh PBS/ethanol was added to the flask containing the LTB_4_-MS and the experiment was continued.

The supernatants were transferred to appropriate vials for determination of the concentration of LTB_4_ by a competition enzyme immunoassay, according to manufacturer's instructions (EIA, Amershan Biosciences, Piscataway, NJ, USA). Quantification was accomplished using calibration curve containing LTB_4_ synthetic standards (Cayman Chemical, Ann Arbor, MI, USA).

### Macrophage cell culture

The J774.1 murine macrophage cell line was obtained from the American Type Culture Collection (ATCC, Rockville, MD, USA). Cells were cultured in Dulbecco′s Modified Eagle′s Medium (DMEM) supplemented with 10% fetal bovine serum (FBS) and 1% Penicilin/Streptomicin (Gibco, Grand Island, NY). After the formation of a monolayer, cells were harvested with plastic cell scrapers and centrifuged at 1,500 rpm for 10 min at 10°C. Next, supernatants were discarded and 10 mL of DMEM was added to each tube of cells. Cell viability and total cell numbers were determined by counting live and dead cells in a Neubauer chamber (BOECO Germany, Hamburg, Germany) after staining with Trypan blue (Gibco). Cells were plated in 96-well culture plates (Cell Wells - Corning Glass Workers) at a density of 1x 10^5^ cells / well and incubated overnight in DMEM in an incubator with a moist atmosphere of 5% CO_2_ at 37° C.

### Treatment with immunostimulatory and pharmacological agents

For osteoclastic cell differentiation, soluble mediator RANKL and monocyte colony stimulating factor (M-CSF / CSF-1) were added to the medium. Briefly, cells were cultured in the presence of M-CSF (30 ng / mL; R&D Systems, Minneapolis, USA), and with RANKL (10 ng / mL; R&D Systems). To confirm the osteoclastic phenotype, the cells were fixed and marked for TRAP (tartrate resistant acid phosphatase). The expression of genes that indicate an osteoclast phenotype was performed by RT-PCR in real time. Then, cells were plated at a density of 1 × 10^5^ cells per well in 96-well culture plates and stimulated with LTB_4_ encapsulated in microspheres at 0.01 µM and 0.1 µM, for 12, 24, and 48. For experimentation, DMEM medium without FBS was used and the plates were kept in an incubator at 37^o^ C and 5% CO_2_.

### Cytotoxicity - Lactate dehydrogenase (LDH) assay

For cytotoxicity assessment, cells were plated in serum-free medium, at a concentration of 1 × 10^5^ cells per well, in 96-well plates and kept in an incubator at 37^o^C and 5% CO_2_ for 12 hours (*overnight*). After this period, cultures were stimulated with different concentrations of pharmacological and immunostimulating agents, for 24 hours. Next, 50 µL of the supernatant was collected and transferred to a new 96-well plate with a transparent, flat bottom and 50 µL of the CytoTox 96® Reagent was added to each sample. The plate was then covered with foil to protect against light and the samples incubated at 25^o^ C for 30 minutes. After this period, 50 μL of the Stop Solution was added to each well. The absorbance was measured at 490 nm with a spectrophotometer (mQuanti, Bio-Tek Instruments, Inc., Winooski, VT, USA). As positive control, 10× Lysis Solution was added to the cells, 45 minutes prior to adding CytoTox 96® Reagent. LDH levels were expressed as percentages, according to the formula: cytotoxicity (%) = 100 × Experimental LDH Release absorbance / Maximum LDH Release absorbance (positive control). Groups were compared using the one-way ANOVA test followed by Dunnett's post-test (α = 0.05).

### In vitro MS uptake by macrophages

Macrophages cells firmly adhered to a cover glass (13 mm) in 24-well plates (2 x 10^5^ cells/well) were incubated for 24h and 48h with 1mL of a suspension of unloaded-MS or LTB_4_-MS (1 mg/mL) in complete RPMI. Medium alone was used as a negative control. After incubation, the non-ingested MS were detached, and the cover glasses were removed from the plates and stained with panoptic stain. In one set of experiments, cytochalasin was used as negative control for phagocytosis. Cells adhering to a cover glass were pre-incubated for 30 minutes with cytochalasin (20 𝛍g/mL) before the addition of MS. MS internalized by macrophages were visualized microscopically and the percentage of cells that ingested at least one MS was calculated. The phagocytic index (PI) was also calculated: PI = number of engulfed MS x number of AMs containing at least one MS/total number of cells ^31^.

### Determination of osteoclast formation by means of the activity of tartrate-resistant acid phosphatase (TRAP) enzyme

Cells were incubated in a solution containing 8 mg of naphthol AS-MX di-sodium phosphate salt (Sigma-Aldrich) in 500 µL of NN-dimethylformamide, followed by the addition of 50 mL of a 0.2 mol / L buffer solution sodium acetate (pH 5.0), containing 70 mg of Fast Red ITR (Sigma-Aldrich). Then the substrate sodium tartrate dihydrate (50 mmol / L) was added to the solution and incubated at 37° C for 2 hours. Subsequently, the wells were washed in distilled water and the cells stained with Harris' hematoxylin. As a control, cells were incubated with medium without the substrate. The analysis of the formation of osteoclasts positive for the TRAP enzyme was determined based on the presence or absence of labeling, in two independent experiments. First, the plates were photographed under bright field microscopy, in 10 × magnification. For quantification of multinucleated TRAP + cells by field of view, the Software Image J (National Institutes of Health, Bethesda, MD, USA) and the image deconvolution plugin (Color Deconvolution) were used. Fast Red vector was applied and then the selected red channel and threshold were manually adjusted. To count the number of cells, the "Analyze Particles” tool was used, after calibration so that the count included only structures with a minimum pixel size of 500, which is equivalent to the size of a multinucleated cell with at least 3 nuclei. The groups were compared using the one-way ANOVA test followed by the Tukey post-test (α = 0.05).

### RNA extraction, reverse transcription, and polymerase chain reaction in real time (qRT-PCR)

mRNA levels were measured by quantitative reverse transcriptase-polymerase chain reactions (qRT-PCR) for samples obtained *in vitro* and *in vivo*. To this end, total RNA was extracted using the RNeasy® Mini kit (Qiagen Inc., Valencia, USA) and quantified using NanoDrop 2000 spectrophotometer (Thermo Fisher Scientific Inc., Wilmington, USA). A total of 1 µg of RNA were used for cDNA synthesis with the High Capacity cDNA Reverse Transcription kit (Applied Biosystems, Foster City, USA) in a thermal cycler (Veriti® Thermal Cycler, Applied Biosystems, USA). qRT-PCR reactions were performed in duplicate using the TaqMan® system in a StepOne Plus® real-time PCR system (StepOne Plus® Real-Time PCR System, Applied Biosystems) and the following cycle program: 95 °C for 20 s, 40 cycles at 95 °C for 1 s, and 60 °C for 20 s. Primer-probe pairs included *Alox5* (Mm01182747), *Alox5ap* (Mm00802100), *Acp5* (Mm00475698), *Mmp9* (Mm00442991), *Ctsk* (Mm00484039) and *Calcr* (Mm01197736) (TaqMan® Gene Expression Assay, Applied Biosystems). All protocols were performed according to the manufacturers’ instructions. Glyceraldehyde-3-phosphate dehydrogenase (*Gapdh*) and beta-actin (*Actb*) were used as reference genes for normalization purposes. The results were analyzed based on cycle threshold (Ct) values. Relative expression was calculated by the ΔΔCt method. Groups were compared using one-way ANOVA test followed by the Tukey post-test or the Dunnett post-test (α = 0.05).

## Results

### 5-LO pathway protects against osteoclastic formation in vivo

**Figure 1:**
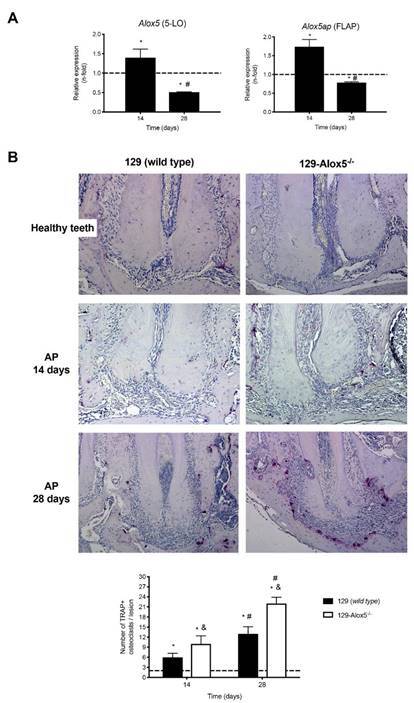
(A) *In vivo* relative expression of *Alox5* and *Alox5ap*, genes which encode 5-LO enzyme and the 5-LO activating protein (FLAP), 14 and 28 days after induction of periapical bone resorption in wild type 129 mice. * p <0.05 compared to gene expression in healthy teeth; # p < 0.05 comparison between periods. (B) Number of osteoclasts positive for the tartrate-resistant acid phosphatase (TRAP^+^) enzyme, 14 and 28 days after induction of periapical bone resorption in wild-type and 5-lipoxygenase (129-Alox5^-/-^) knockout mice. *p < 0.05 compared to the number of TRAP^+^ osteoclasts in teeth without apical periodontitis (dashed line), ^&^ *p* < 0.05 compared to wild-type mice, ^#^ *p* < 0.05 comparison between periods to the same group of mice. Right panel depicts photomicrography at 10 and 20x.

During apical periodontitis development, synthesis of genes that encode 5-LO (*Alox5*) and 5-lipoxygenase activating protein (FLAP; *Alox5ap*) were upregulated at 14 days (p <0.05) and down regulated latter on (p <0.05; Figure 1). This pattern of 5-LO pathway gene expression was different from osteoclast recruitment periapically, once wild type mice showed an increased osteoclast formation overtime. Interestingly, both at 14 and 28 days, the number of osteclasts found in periapical bone resorption in 5-LO knockout mice was higher than that found in wild type mice (p <0.05). These results demonstrated that the 5-LO pathway and the metabolites produced during the arachidonic acid metabolism impaired the osteoclast differentiation process.

### Characterization of microspheres prepared for sustained delivery of LTB_4_

No bacterial contamination was found in any batch of prepared microspheres, after culture in BHI-agar medium, for a period of 24 hours in an incubator. Likewise, no endotoxin was detected in the samples of each prepared batch. Therefore, empty microspheres or containing encapsulated LTB_4_ were used in subsequent experiments. The encapsulation efficiency of LTB_4_ was 38 ± 2.2%.

After lyophilization of the microspheres obtained by the solvent evaporation method, the characterization was carried out by dispersing their diameters, after reconstitution in distilled and deionized water. The average diameter of the empty microspheres was 4.1 ± 2.7 μm and the microspheres containing LTB_4_ (MS-LTB_4_) was 5.1 ± 4.5 μm. Therefore, the microspheres did not have their diameters changed by the LTB_4_ encapsulation process.

Regarding the electrical stability of the microspheres, there were no relevant differences between the means of the zeta potential of the empty microspheres (-21.7mV ± 5.5 mV and of the microspheres containing LTB_4_ (MS-LTB_4_) (-10.7mV ± 3.6 mV). Therefore, LTB_4_ encapsulation did not result in changes in the electrical charge on the PLGA surface.

Shape and topography of the microspheres were determined using scanning electron microscopy. Overall, the microspheres empty or containing LTB_4_ (MS-LTB_4_) presented spherical shape, uniform surfaces and without pores.

### MS-LTB_4_ are not cytotoxic and are engulfed by macrophages

Because we observed a negative modulation of the 5-LO pathway during osteoclastogenesis *in vivo*, we used LTB_4_ in two different concentrations (0.01 μM and 0.1 μM), incorporated in microspheres of lactic acid-co-glycolic acid (PLGA). This strategy was used because the osteoclast differentiation experiment is long (12 to 48 hours) and the half-life of the lipid mediators is short. Thus, we aimed to keep LTB_4_ available for the entire maintenance period of the cell culture.

**Figure 2:**
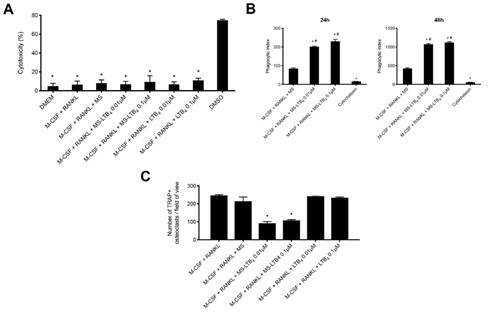
(A) Cytotoxic effect of 0.01 μM and 0.1 μM of LTB_4_ encapsulated in microspheres and empty microspheres, as well as M-CSF + RANKL stimuli, in culture of murine macrophages, measured by through the LDH test, 24 hours after exposure. * p <0.05 compared to the positive control (DMSO). (B) Phagocytic index of macrophages incubated with LTB4-MS. The phagocytic index was calculated after 24 h and 48 h incubation with MS. Pre-treatment of cells with cytochalasin demonstrated that internalization of MS was by cytoskeleton-dependent phagocytosis. * p <0.05 compared to negative control (medium); # p< 0.05 compared to cytochalasin. (C) . Quantification of the number of osteoclasts (TRAP + cells), cultured with M-CSF (30 ng / mL) + RANKL (10 ng / mL), with M-CSF (30 ng / mL) + RANKL (10 ng / mL) containing microspheres (MS) or soluble LTB_4_ in concentrations of 0.01 μM and 0.1 μM, and with M-CSF (30 ng / mL) + RANKL (10 ng / mL) containing empty microspheres (MS), 48 hours after adding the stimuli.* p <0.05 compared to treatment with RANKL and M-CSF.

Microspheres were not cytotoxic to macrophages, regardless of whether they contained LTB_4_ or not, compared to DMSO (positive control; p <0.05). The stimulation with M-CSF + RANKL was not cytotoxic compared to the DMEM culture medium (p> 0.05) (Figure 2A).

After determining the number of cells that phagocytized at least one MS, the phagocytic index was calculated by counting the number of MS inside the cells after 24, and 48 h of incubation. LTB_4_-MS were more efficiently phagocytized than unloaded-MS at both time points evaluated. To analyze only internalized MS and exclude those that were attached to the cell surface, macrophages were pre-treated with cytochalasin, which prevents phagocytosis. Cytochalasin treatment significantly reduced the uptake of unloaded-MS and LTB_4_-MS (Figure 2B).

### MS-LTB_4_ embedded in microspheres inhibits osteoclastogenesis

After 48 hours, treatment with M-CSF + RANKL led to the formation of cell colonies and differentiation of TRAP positive osteoclasts, differently from what was observed in the group maintained with medium alone. Interestingly, treatment with M-CSF + RANKL + LTB_4_ microspheres reduced osteoclast formation on the culture plate (p < 0.05), regardless of the concentration used. Treatment with M-CSF + RANKL + empty microspheres or soluble LTB_4_ did not prevent osteoclast formation (p> 0.05). These results show that LTB_4_, a metabolite of the 5-LO pathway produced by the action of the leukotriene A4 hydrolase enzyme on LTA_4_, reduces the osteoclastogenic potential induced by M-CSF + RANKL (Figure 2C).

### LTB_4_-MS prevents *Acp5*, *Mmp9*, *Calcr* and *Ctsk* gene expression

In order to investigate the effect of exogenous addition of LTB_4_ on the synthesis of molecules involved in osteoclast differentiation, the gene expression of a panel of markers of osteoclast formation and activity was evaluated.

**Figure 3:**
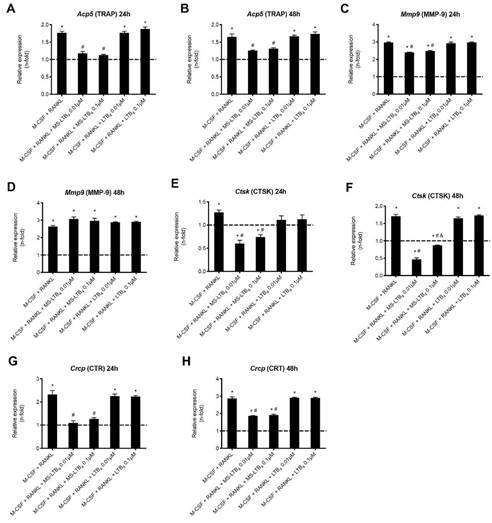
Relative expression of the *Acp5* (A, B), *Mmp9* (C, D), *Ctsk* (E, F) genes and *Calcr* (G, H) that encode the TRAP, matrix metalloproteinase-9, cathepsin K and calcitonin receptor respectively. RNA was extracted 24 and 48 hours after stimulation of murine macrophages with M-CSF (30 ng / mL) + RANKL (10 ng / mL) containing soluble LTB_4_ or incorporated in microspheres (MS) at concentrations of 0.01 μM and 0.1 μM. * p <0.05 compared to expression in cells maintained in DMEM without FBS (dashed line); # p <0.05 compared to adding M-CSF + RANKL only; & p <0.05 comparing the different LTB_4_ concentrations.

*Acp5* gene, which encodes the TRAP enzyme, was induced by the addition of M-CSF + RANKL and treatment with LTB_4_-MS for 24 or 48 hours after the stimulus prevented *Acp5* expression (p< 0.05) (Figure 3A and 3B). *Mmp9* gene, which encodes matrix-9 metalloproteinase enzyme, was stimulated by the addition of M-CSF + RANKL to the culture medium, both 24 and 48 hours after stimulation (p <0.05). Interestingly, after 24 hours LTB_4_-MS reduced *Mmp9* expression whereas after 48 hours it had no effect on *Mmp9* synthesis (Figure 3C and 3D).

*Ctsk* gene, which encodes the enzyme cathepsin K, was induced by adding M-CSF + RANKL to the culture medium (p< 0.05), and the expression decreased after adding LTB_4_-MS (p < 0.05), both at 24 and 48 hours. The effect of LTB_4_-MS on *Ctsk* expression was higher using the 0.1 μM concentration at 48 hours (p < 0.05) (Figure 3E and 3F). *Calcr* gene, which encodes the calcitonin receptor, was induced by the addition of M-CSF + RANKL and treatment with LTB_4_-MS for 24 or 48 hours after the stimulus prevented *Acp5* expression (p< 0.05). (Figure 3G and 3H).

## Discussion

Modulation of the 5-LO proinflammatory pathway was observed *in vitro* during hematopoietic cell differentiation into osteoclasts and the addition of LTB_4_-containing microspheres inhibited the osteoclastogenic potential of the culture induced by the soluble mediator RANKL. These results corroborate with our current and prior *in vivo* observations that this pathway plays a protective role during *in vivo* osteoclastogenesis ^13^ ^,^ ^15^.

Inflammatory process in the face of aggressive microbial stimuli is a response from the host that aims to fight infection and restore its physiological functioning. Cytokines and various signaling molecules are synthesized and secreted by resident cells, prior to the recruitment and activation of cells of the immune system, generating a molecular pattern of immune response. In this process, lipid mediators derived from arachidonic acid are important molecules in the regulation of homeostatic and inflammatory processes ^23^.

In bone tissue, there is a constant interrelation between the inflammatory response and the resorption of mineralized matrices. Thus, considering that we observed a modulation of the 5-LO pro-inflammatory pathway during osteoclastogenesis and that a previous study showed that LTC_4_ is important for osteoclastic differentiation of bone marrow-derived macrophages ^4^, we investigated whether LTB_4_ added exogenously could modify the course of cell differentiation and clarify a possible mechanism involved in osteoclast formation in animals deficient of the 5-LO enzyme.

Microspheres were used as the delivery system for LTB_4_ in this study. This encapsulation method allows LTB_4_ to maintain its biological activity preserved, in addition to being an important tool for cellular stimulation, in order to protect the encapsulated mediator from degradation ^22^. After evaluation for a period of 24 hours, it is known that microspheres of LTB_4_ had a peak release of 45% of the mediator in a period of 5 hours, and can be continuously released for a long period of time ^21^. The microspheres were prepared using a polymer, the co-glycolic poly-lactic acid (PLGA). PLGA is widely used for preparation of microspheres because it is a biodegradable and biocompatible polymer ^24^ ^,^ ^25^. This material allows the release of encapsulated molecules in a controlled manner for long periods and from a single administration, which keeps the concentrations of the encapsulated substance constant ^26^.

LTB_4_ has been efficiently encapsulated in PLGA previously ^21^ ^,^ ^22^, without prejudice to its biological activity. This polymer has high encapsulation efficiency, stability and its degradation products (lactic and glycolic acid) are also biocompatible ^27^. Specifically with respect to LTB_4_, this delivery system proves to be a relevant alternative to conventional drug delivery systems, since in addition to releasing the encapsulated molecules in a controlled manner over longer periods through a single administration, it maintains the stability of mediators extremely labile ^25^ ^,^ ^26^. Lipid mediators can be easily degraded, via oxidation and hydrolysis reactions, and consequently lose their biological properties. Therefore, encapsulation is an efficient way to maintain the constant concentration of lipid mediators, in order to prevent its degradation.

In a previous study, LTB_4_ induced RANKL expression, which in turn led to osteoclast differentiation ^28^. In the absence of metabolites from the 5-LO pathway, there was a reduction in recruitment and differentiation of osteoclastic cells, preventing bone resorption induced by mechanical load ^29^. However, a comparison of these studies regarding the osteoclastogenic potential of LTB_4_ should consider some factors. The first is that this modulation can be modified by the presence or absence of concomitant infection, as well as by the concentration of the mediator used. The second, and perhaps more relevant, is the delivery system used. Up to date, the role of LTB_4_ in osteoclastogenesis was evaluated using the mediator in the soluble form ^4^ ^,^ ^16^ ^,^ ^28^ ^,^ ^30^, which acts mainly on the BLT1 and BLT2 surface receptors. We used the encapsulation in PLGA microspheres as a strategy. Encapsulation favors the engulfment of microspheres by phagocytes such as macrophages J774A.1 ^27^, which allows this mediator to be released inside the cell and signal via nuclear receptors of the PPAR family. Polymeric microspheres containing LTB_4_ are more phagocytized by murine peritoneal macrophages than empty microspheres ^21^ ^,^ ^22^ and are capable of inducing an increase in the production of nitric oxide in human endothelial cells and in peritoneal macrophages murine, and increase the expression of the nuclear receptor PPAR-α ^22^ ^).^ Indeed, PPAR agonists treatment of osteoclasts derived from human CD14+ monocytes reduced the activation of RANKL signalling pathways and expression of key osteoclast genes ^31^. One limitation of this study is that this mechanism was not demonstrated *in vitro*, albeit it can be hypothesized that the dual role of the lipid mediator might be dependent on the family of receptors that are activated in the signaling cascade. This subject deserves further investigation.

Stimulation of human mononuclear cells with M-CSF associated with LTB_4_ increased the expression of osteoclastogenesis marker genes such as *CTSK*, *MMP9* and *ACP5* ^16^ *,* differently from what we observed with an immortalized culture of macrophages obtained from mice, in which LTB4 induced the expression of *Mmp9* but inhibited *Calcr* and *Ctsk*, without changing *Acp5*. Inhibition of 5-LO might induce the anabolism ^4^ ^,^ ^13^ ^,^ ^15^ or catabolism ^32^ in bone remodeling. LTB_4_ induce RANKL and osteoclast differentiation of bone marrow-derived and RAW-264.7 ^16^ ^,^ ^29^ ^,^ ^30^ but not J774.1 macrophages. Aside the cell line used for experimentation, these divergent results could be due to the incorporation of LTB_4_ in PLGA microspheres and, therefore, shed light to a novel protective role of the lipid mediator as inhibitor of osteoclast differentiation.

## Conclusion

Leukotriene B_4_ incorporated into microspheres inhibited differentiation of macrophages into an osteoclastic phenotype and activation of the cells under RANKL stimulus.
